# Recent Research on Febrile Seizures: A Review

**DOI:** 10.4172/2155-9562.1000165

**Published:** 2013-09-25

**Authors:** DO Syndi Seinfeld, John M. Pellock

**Affiliations:** Department of Neurology, Virginia Commonwealth University, Richmond, USA

## Abstract

Febrile seizures are common and mostly benign. They are the most common cause of seizures in children less than five years of age. There are two categories of febrile seizures, simple and complex. Both the International League against Epilepsy and the National Institute of Health has published definitions on the classification of febrile seizures. Simple febrile seizures are mostly benign, but a prolonged (complex) febrile seizure can have long term consequences. Most children who have a febrile seizure have normal health and development after the event, but there is recent evidence that suggests a small subset of children that present with seizures and fever may have recurrent seizure or develop epilepsy. This review will give an overview of the definition of febrile seizures, epidemiology, evaluation, treatment, outcomes and recent research.

## Introduction

Febrile seizures (FS) are the single most common seizure type and occur in 2 to 5% of children younger than age 5 years with a peak incidence in the second year of life [1]. Historically FS were studied using large epidemiologic studies. Initial studies did not exclude seizures associated with underlying neurological disturbance [2], and prognosis was pessimistic because of the inclusion criteria [3]. It is currently accepted that most children who have a FS often have normal health and development after the event. FS are considered benign, but there is recent evidence that suggests a small subset of children that present with seizures and fever may have recurrent FS or develop epilepsy.

The incidence and prevalence of FS is similar across the numerous FS studies. There is variation of incidence of FS based on geographic location, with higher prevalence found in Japan and Guam [4–6]. FS are not considered a form of epilepsy, but a FS can be the first presentation of subsequent epilepsy. At this time it is not possible to predict which child will develop an a febrile seizure after presenting with FS [7]. This review will give an overview of the definition of FS, epidemiology, evaluation, treatment, outcomes and recent research.

## Definition

The International League against Epilepsy (ILAE) defines a FS as a seizure occurring in childhood after one month of age, associated with a febrile illness that is not caused by an infection of the central nervous system. A child with the diagnosis of FS cannot have a history of neonatal seizures, a previous unprovoked seizure or meet criteria for other acute symptomatic seizures [8]. The lower age limit of the ILAE definition is younger than the limit proposed previously by the National Institutes of Health (NIH). The NIH Consensus Conference definition of FS is an event usually occurring between 3 months and 5 years of age, associated with fever, but without evidence of intracranial infection or defined cause [9].

FS can be separated into two categories, simple and complex. A simple febrile seizure is isolated, brief and generalized [10]. Complex FS is one with focal onset, one that occurs more than once during a febrile illness, or one that lasts more than 10 to 15 minutes [11]. Developmental delay and younger age are associated with prolonged FS [12]. Subsequent febrile seizures can be prolonged if the initial febrile seizure was prolonged [13]. Febrile status epilepticus (FSE) is a subgroup of complex FS.

## Epidemiology

FS have a peak incidence at 18 months of age and are most common between 6 months and 5 years [10]. Most FS are simple with approximately 20–30% being complex [13]. The distribution of a first FS duration can be described using a two population model, one with short seizure duration and the other with long seizure duration, with the cut-off at approximately 10 minutes [12]. Approximately 5% of FS will last ≥30 minutes [14]. No correlation has been identified between duration of the first FS and duration of the second FS [12]. Although, it has been observed that a recurrent febrile seizure is more likely to be prolonged if the initial FS was prolonged [13].

By definition, a febrile illness is required for a child to have a FS. Children with FS have higher temperatures with illness compared to febrile controls [15]. The rapid onset of fever was previously thought to be precipitating factor of FS, but this is no longer thought to be true [16]. Gender predominance of FS has also been studied. There are studies that conclude a higher incidence of FS in males [17] and others showed no significant difference based on gender [18].

FS occur in the setting of a febrile illness, which could cause seasonal variation. In Japan a study of FS showed two peaks of incidence, November to January and June to August, which correspond to peaks of viral upper respiratory infections and gastrointestinal infections respectively [19]. A study performed in Italy, which looked at 188 first FS, found that there is a significant increase in FS from 6 PM to 11:59 PM and a seasonal peak in January [20]. There have been multiple studies have supported the conclusion that FS have a peak in the winter and end of the summer [21–23]. Influenza A has been found to have a significant relationship with recurrence of FS [24]. Although it has been suggested that FS are more likely to occur with respiratory illnesses compared to viral gastrointestinal illness [15,25], any febrile illness can cause a FS. For example, in malaria endemic regions there is an increased incidence of FS when there is increased malaria transmission [26].

## Provoking Factors

### Genetics

FS can be seen in multiple family members and there is evidence of genetic and environmental causes. There is a variable inheritance pattern, with no single accepted mechanism. A positive family history of FS can be found in 25–40% of cases when a child present with a FS [27–29]. The number of FS a child has affects the risk of a sibling experiencing a FS [30]. Significantly higher concordance rates are seen for FS in monozygotic twins as compared to dizygotic twins in multiple twin registries [31].

The phenotype of FS plus may account for children without a specific epilepsy syndrome who have FS and then developed generalized epilepsy [32]. These patients or family members have a have a history of FS, often complex and frequently occur beyond 5 years of age. Epilepsy with variable seizure types develop later in childhood or adulthood. A variety of mutations including SCN1A, SCN1B, and GABGR2 have been demonstrated in these families [33]. The proposed genetic syndrome that is called generalized epilepsy with febrile seizures plus (GEFS+) is a spectrum of clinical epilepsy phenotypes, with the most severe phenotype of myoclonic-astatic epilepsy [32].

### Intrauterine risk factor

A population-based, prospective questionnaire study from early fetal life onward evaluated the occurrence of FS in 3,372 subjects at age 12 and 24 months [34]. Children in the lowest percentile of transverse cerebellar diameter in the second trimester were at increased risk of developing FS, compared with children in the highest percentile. In the third trimester, children in the lowest percentile of all general growth characteristics (femur length, abdominal circumference, and estimated fetal weight) were at increased risk of developing FS. Children in the lowest percentile of biparietal diameter in the third trimester also were at increased risk of FS. The study concluded fetal growth retardation is associated with increased risk of FS and that adverse environmental and genetic factors during pregnancy may be important in the development of FS. Furthermore, the Danish birth cohorts (Aarhus Birth Cohort, Aalborg-Odense cohort and the Danish National Birth Cohort) have demonstrated that low birth weight and short gestational age are significant risk factors for FS [35].

### Vaccinations

Vaccinations are important to pediatric health and are recommended by the American Academy of Pediatrics (AAP) for members of the youngest age group at risk for experiencing the disease for which efficacy and safety have been demonstrated [36]. The World Health Organization (WHO), in 2002, released a publication on immunization safety. WHO recommends that children be vaccinated. In general, FS that occur after vaccination have not been found to be different from FS from other causes [37]. Historically, there were a group of children that were thought to have severe FS and encephalopathy from vaccine induced FS. The children presented with a first FS after vaccination and developed encephalopathy and recurrent seizures, but it is known that this subgroup have a genetic sodium channel mutation that causes a susceptibility to severe seizures and encephalopathy. The FS is commonly the first manifestation of the sodium channel mutation known as Dravet syndrome, but any febrile illness could cause the first seizure in a genetically susceptible individual. Vaccinations can trigger the onset of seizures in one third of patients with Dravet syndrome [37]. FS will not cause an epileptic encephalopathy in a child without a mutation conferring genetic susceptibility. Neither the AAP nor WHO recommend stopping or changing the immunization schedule after a FS, even in children with underlying genetic mutations.

The public fear of vaccines causing FS has led to numerous studies. Children less than 2 years of age have an increased risk of FS after the first dose of a measles containing vaccine when it is administered with a varicella vaccine [38]. The measles containing vaccines have not been found to be associated with an increased risk of FS in children over 4, regardless of whether varicella is given at the same time [39]. The previously used whole-cell diphtheria/tetanus/pertussis and measles-containing vaccines have an established association with FS, but the less reactogenic diphtheria, tetanus, and a cellular pertussis (DTaP) vaccine has been developed and is currently used and does not increase the risk of FS [37]. There is no evidence that children should not be vaccinated.

### Metabolic abnormalities and deficiencies

Whereas some have reported a statistical association between iron deficiency anemia and simple febrile seizures [40,41], other cross sectional studies have not found a significant association [42]. A study of febrile seizures in Indian children found lower zinc levels in patients with FS compared to age matched febrile children without seizures [43]. Other studies have proposed that there is a link between FS and a systemic respiratory alkalosis, irrespective of the severity of the underlying infection [44]. These associations require large population studies to determine if they can be predictive or if they can ultimately be factors associated with prevention.

## Evaluation

The AAP has guidelines for evaluation of first simple febrile seizure, and state that clinicians should work to identify the source of the fever when a child presents within 12 hours of a simple febrile seizure [45]. There have been recent US studies to suggest that the risk of bacterial meningitis presenting as a first simple FS is very low and that the above guidelines are not being strictly followed [46,47]. Alternatively, in countries with high prevalence of diseases, such as malaria, these guidelines for evaluation cannot be as easily followed. Children with malaria who develop fever frequently have complex FS, and it is often difficult to differentiate children with intracranial infection from those without [48].

### Laboratory studies

It is not recommended to perform routine serum labs when a child has a simple febrile seizure [49]. The labs that are performed should be based on the clinical presentation of the febrile illness.

The Consequences of Prolonged Febrile Seizure Study (FEBSTAT), which enrolled 199 children from 1 month to 5 years of age with FSE, evaluated children for the presence of HHV-6A, HHV-6B, or HHV-7 DNA and RNA in serum [50,51]. Children that had central nervous system infection were excluded from this study, thus no subject had encephalitis. The study concluded HHV-6B infection is commonly associated with FSE, and HHV-7 infection is less frequently associated with FSE. Together the HHV infections accounted infection in one third of FSE subjects in the study [50]. The study did not detect HHV-6B or HHV-7 DNA in the CSF of the 23 subjects who presented in FSE with documented HHV-6B or HHV-7 viremia.

### Lumbar puncture

The AAP guidelines strongly recommend a lumbar puncture in any child who presents with a seizure, a fever and has meningeal signs and symptoms. It is also recommended in any child whose history or examination suggests the presence of meningitis or intracranial infection [45]. As part of the FEBSTAT study, cerebrospinal fluid (CSF) samples were reviewed for the 136 of the 199 children who had a nontraumatic lumbar puncture performed. The study confirmed that FSE rarely causes CSF pleocytosis [52]. The CSF glucose and protein levels of the sample were unremarkable, and the temperature, age, seizure focality, and seizure duration did not affect results. This supports that CSF pleocytosis should not be attributed to FSE.

### EEG

There is no evidence that an EEG can be used to predict if a child will develop epilepsy after a simple febrile seizure. A clinician should consider performing EEG if more than one complex feature is present [53], but a routine practice of obtaining an early EEG in neurologically normal children with complex febrile seizures was not initially justified [54]. EEG does have a role if a child remains encephalopathic after a FS.

The FEBSTAT study performed baseline EEGs within 72 hours of the episode of FSE. Review of the baseline EEGs showed that there is focal EEG slowing or attenuation in a substantial proportion of children [55]. The slowing and attenuation are highly associated with MRI evidence of acute hippocampal injury [55]. These findings may be a sensitive and readily obtainable marker of acute injury associated with FSE.

### Imaging

Neuroimaging is not recommended after a simple febrile seizure [49]. If a patient presents with focal complex FS and/or FSE, one should consider performing brain MRI to evaluate for a structural abnormality as an explanation for the seizure [51]. A head MRI is more sensitive for abnormalities that can cause seizures, but when unavailable computed tomography can be performed.

The FEBSTAT study included baseline imaging on recruited subjects [51]. One hundred ninety one of the children had baseline MRI of the brain with emphasis on the hippocampus. A total of 22 children had definitely abnormal or equivocal increased T2 signal in the hippocampus following FSE [56]. None of the children in the control group had this abnormality, which was statistically significant [56]. The imaging also showed that developmental abnormalities of the hippocampus were more common in the FSE group than in controls, with hippocampal malrotation being the most common. This study has demonstrated that children with FSE are at risk for acute hippocampal injury and that a substantial number also have abnormalities in hippocampal development. These cohorts of FSE subjects are still being followed to determine the long-term outcomes in these children.

## Treatment

### Acute

#### Simple FS

**Figure 1:**
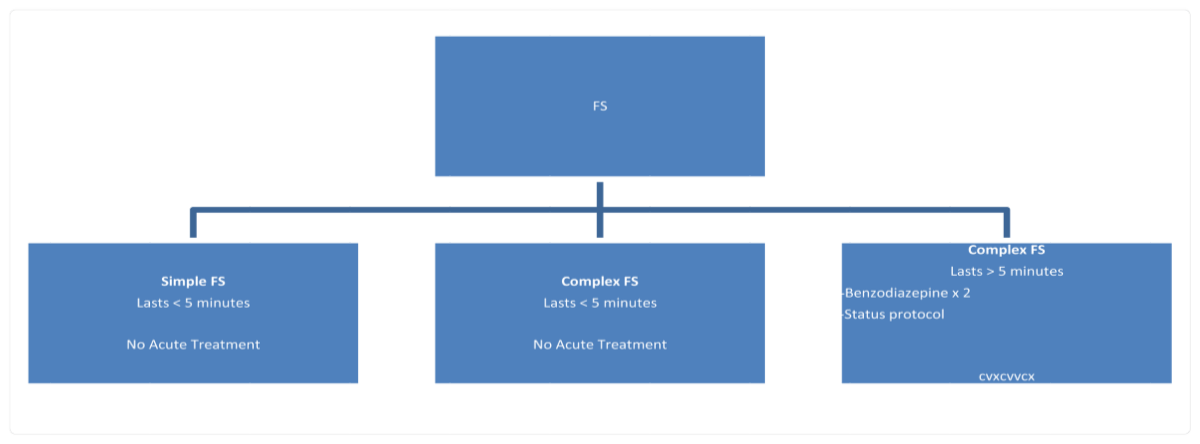
Treatment of FS based on seizure duration.

There is no evidence that treatment of simple febrile seizures can prevent later development of epilepsy [57] and thus is not recommended (Figure 1).

#### Complex FS

Prolonged febrile seizures, lasting more than 10 minutes, are unlikely to stop spontaneously [51]. A child that is actively having a seizure should receive acute abortive treatment after 5 minutes of the seizure starting. Pediatric status epileptics (SE) have traditionally been defined as a seizure that lasts for more than 30 minutes. This operational definition has been revised. More recent articles and studies have advocated that the duration of pediatric SE should be operationally shortened to seizures lasting more than 5–10 minutes [58,59]. Prolonged seizures are accompanied by an increased risk of complications, and treatment should be initiated before it reaches 10 minutes in duration. Prolonged FS should be treated acutely using the same algorithm as prolonged seizures caused by other etiologies. A prolonged FS should be treated acutely by emergency medical services (EMS) or the emergency department. Initial use of a benzodiazepine is recommended, and if continued seizure then a full SE protocol should be initiated [10]. FSE is a neurological emergency and the most common cause of SE in children younger than two years of age [60].

### Chronic

Using around-the-clock prophylactic administration of antipyretics has not been shown to affect the incidence of recurrence of FS, and is not recommended [61]. It is not recommended to treat children with FS using daily anti-epileptic medications because of high likelihood of adverse effects [62].

## Recurrence

The risk of recurrence is influenced by both the age of the child and the type of FS. About one-third of children with a first FS will have a recurrence. Risk factors for recurrence include family history of FS, less than 18 months of age, temperature lower than 40.0°C at first convulsion and less than 1 hour between onset of febrile illness and first convulsion [10,62,63]. The numbers of risk factors are directly proportional to the risk of recurrence. A child with two or more risk factors has a more than 30% recurrence risk at 2 years of age, and that risk doubles with three risk factors [61] (Table 1).

## Differential Diagnosis

There are seizures in the setting of fever that are not considered FS. For example, falciparum malaria is a cause of acute symptomatic seizures in children admitted to hospitals in sub-Saharan Africa, and these seizures are associated with neurological disabilities and epilepsy [64]. These children may have fever, but the seizures are a different category compared to FS. Dravet syndrome, a severe epilepsy syndrome, can present with early febrile seizures, but the seizures progress and become a febrile and intractable [65]. These children initially present as FS, but are not true FS. Mitochondrial disease can present similarly to Dravet syndrome, with early febrile seizures and progression to developmental regression, ataxia, behavior change and a febrile seizures [65]. When a child has an underlying metabolic or mitochondrial disease they may have a seizure associated with a febrile illness, but they would not be classified as FS. It may be difficult to determine if there is an underlying disorder with an initial FS, but the progressive course would indicate different etiology of seizure.

## Outcome

There is evidence that FS are associated with an increased risk of subsequent epilepsy, and that epilepsy develops in 2 to 4% of children with a history of FS [18]. Although it is accepted that a single brief simple FS is benign with no clinical consequences, the risk of developing epilepsy can be as great as 57% in children with focal, prolonged, and recurrent FS [28].

A prospective cohort study performed using children presenting with first FS observed that developmental delay is associated with prolonged FS [12]. There are greater frequencies of delays in reaching developmental motor milestones at baseline in children with long versus short FS. Thus, children with prolonged FS are more likely to be neurologically abnormal than children with single, short, nonfocal FS [13].

Additionally, having had more than one complex feature of a febrile seizure further increased the risk of developing subsequent unprovoked seizures [66]. Risk factors for developing epilepsy after febrile seizure include neurodevelopmental abnormality, complex FS, family history of epilepsy and duration of fever [10] (Table 2). There is no evidence that preventing FS prevents the development of epilepsy.

## Current Research

The National Institute of Neurological Disorders and Stroke (NINDS), which is part of the National Institutes of Health (NIH), sponsors febrile seizure research in the United States, but FS are the focus of numerous current studies worldwide. There are basic science studies that are using a rat model to evaluate neuronal injury from FS, and looking at future development of epilepsy following FS [67]. The North London Status Epileptics in Childhood Surveillance Study (NLSTEPSS) in the United Kingdom is evaluating incidence, morbidity and treatment children with SE, including FSE [68]. The FEBSTAT study is evaluating the long-term consequences of FSE using MRI, EEG, developmental/neuropsychological testing, virology, genetics, psychiatric interview and parental interviews [51]. The genetics of FS, the sub-types and sub-syndromes are also being studied using twin pairs [69].

## Summary

FS are common and mostly benign. Unfortunately, FSE is frequently not recognized and prolonged seizures frequently need medication to terminate the seizure [51]. Fortunately, studies have found that a simple FS is mostly benign, but prolonged febrile seizures do have long term consequences. They should be treated acutely if they continue for past 5 minutes. It is important to educate parents about the risks associated with febrile seizures and the at home treatments for prolonged febrile seizures (ex. rectal diazepam). Education about seizure safety and precautions should be explained to the family, and are important to prevent consequences of prolonged seizures.

## Figures and Tables

**Table 1:** Risk factors for recurrence of febrile seizures.

1. family history of FS
2. age less than 18 months
3. temperature lower than 40.0°C at first convulsion
4. less than 1 hour between onset of febrile illness and first convulsion

**Table 2:** Risk factors for developing epilepsy after febrile seizures.

1. neurodevelopmental abnormality
2. complex FS, including FSE
3. family history of epilepsy
4. duration of fever
